# Machine-learning-based diagnosis of drug-naive adult patients with attention-deficit hyperactivity disorder using mismatch negativity

**DOI:** 10.1038/s41398-021-01604-3

**Published:** 2021-09-18

**Authors:** Sungkean Kim, Ji Hyun Baek, Young Joon Kwon, Hwa Young Lee, Jae Hyun Yoo, Se-hoon Shim, Ji Sun Kim

**Affiliations:** 1 Department of Human-Computer Interaction, Hanyang University, Ansan, Republic of Korea; 2 Department of Psychiatry, Samsung Medical Center, Sungkyunkwan University School of Medicine, Seoul, Republic of Korea; 3 Department of Psychiatry, Soonchunhyang University Cheonan Hospital, Cheonan, Republic of Korea; 4 Department of Psychiatry, Seoul St. Mary’s Hospital, College of Medicine, The Catholic University of Korea, Seoul, Republic of Korea

**Keywords:** Diagnostic markers, Psychiatric disorders

## Abstract

Relatively little is investigated regarding the neurophysiology of adult attention-deficit/hyperactivity disorder (ADHD). Mismatch negativity (MMN) is an event-related potential component representing pre-attentive auditory processing, which is closely associated with cognitive status. We investigated MMN features as biomarkers to classify drug-naive adult patients with ADHD and healthy controls (HCs). Sensor-level features (amplitude and latency) and source-level features (source activation) of MMN were investigated and compared between the electroencephalograms of 34 patients with ADHD and 45 HCs using a passive auditory oddball paradigm. Correlations between MMN features and ADHD symptoms were analyzed. Finally, we applied machine learning to differentiate the two groups using sensor- and source-level features of MMN. Adult patients with ADHD showed significantly lower MMN amplitudes at the frontocentral electrodes and reduced MMN source activation in the frontal, temporal, and limbic lobes, which were closely associated with MMN generators and ADHD pathophysiology. Source activities were significantly correlated with ADHD symptoms. The best classification performance for adult ADHD patients and HCs showed an 81.01% accuracy, 82.35% sensitivity, and 80.00% specificity based on MMN source activity features. Our results suggest that abnormal MMN reflects the adult ADHD patients’ pathophysiological characteristics and might serve clinically as a neuromarker of adult ADHD.

## Introduction

Attention-deficit/hyperactivity disorder (ADHD) has been defined as a neurodevelopmental disorder with symptoms of distracted attention, hyperactivity, and impulsivity [1]. Although ADHD is known to be a disease with childhood-onset, ~15% of children with ADHD maintain their symptomatology, meeting full diagnostic criteria for adults [2]. Detection of adult patients with ADHD is crucial to providing proper management because they still display marked difficulties in maintaining selective attention to relevant information and increased distractibility towards irrelevant stimuli [3, 4]. However, ADHD in adulthood is often misdiagnosed as anxiety or mood symptoms in a clinical setting [5]. Despite the existence of adulthood ADHD and its clinical importance, relatively little is known about the neurobiological background of adult ADHD [6].

Event-related potentials (ERPs) have been widely utilized to study the attentional processes in childhood ADHD [7]. In particular, mismatch negativity (MMN), an ERP component, represents pre-attentive auditory processing that is closely correlated with cognitive status in the absence of behavioral responses as well as motivation [8–10]. Given that patients with ADHD suffer from proper distribution of attention, such as focusing and maintaining their attention on the tasks, MMN has been considered a good neuromarker for evaluating the neurophysiological mechanisms of ADHD. Moreover, MMN reflects glutamatergic function [11], and the critical pathology for MMN reduction might be associated with the N-methyl-D-aspartate (NMDA) receptor system dysfunction [12]. NMDA receptors have been well studied for their crucial role in cognitive function, such as learning and memory [13, 14]. In addition, recent reports also suggest the dysfunction of NMDA receptors in the pathophysiology of ADHD [15]. In this regard, MMN might be a promising biomarker of ADHD.

Several studies have assessed MMN changes and their correlates with attentional problems in children with ADHD [16–20]. A recent meta-analysis revealed a reduced MMN amplitude in children with ADHD compared to healthy controls (HCs) [7]. However, to date, no study has investigated MMN changes in adult ADHD. Regarding maturational processes of brain activity with aging [21], the neurobiological basis of adult ADHD might be different from that of childhood ADHD. In addition, even though the symptoms of adult ADHD are similar to those of childhood ADHD, symptom severity, especially hyperactivity, may decrease over time [22]. This suggests that adults with ADHD may display different MMN responses from those of childhood ADHD.

Recently, an increasing number of researchers have attempted to differentiate patients with ADHD from HCs on the basis of machine learning methods with EEG biomarkers. However, few studies have investigated the classification of adult patients with ADHD and HCs. They have reported accuracies ranging from hardly above chance level (50%) to beyond 90%. For example, Mueller et al. [23, 24] classified adult ADHD patients and HCs using Go-NoGo ERPs and achieved accuracies above 90%. Tenev et al. [25] measured resting-state conditions and neuropsychological tasks, obtaining an accuracy of 82%. Studies of resting-state band power reported 68% accuracy [21] and 76% accuracy [26]. In addition, Kiiski et al. [6] demonstrated that resting-state connectivity features did not reliably classify ADHD patients and HCs.

Detection of electrophysiological markers to differentiate adult ADHD patients could support clinicians to provide appropriate diagnosis and treatment for patients suffering from difficulties in directing and maintaining attention in their lives. However, given that there have been limited studies of adult ADHD, further studies are warranted to identify biomarkers for adult ADHD using machine learning techniques. Moreover, research differentiating between adult patients with ADHD and HCs using MMN features has not yet been undertaken.

The aim of this study was to investigate changes in MMN features in adult patients with ADHD compared to HCs at both the sensor (amplitude and latency) and source levels (source activation). In addition, we explored the relationships between MMN features and ADHD symptom scores. Finally, we examined the possibility of MMN features serving as biomarkers by differentiating between ADHD patients and HCs using machine learning techniques. We hypothesized that MMN activities at both the sensor and source levels would be attenuated in adult ADHD patients compared to HCs, reflecting the pathophysiology of ADHD. We also hypothesized that these MMN characteristics could differentiate between ADHD patients and HCs with acceptable classification performances. To the best of our knowledge, this study is the first to examine the differences in MMN characteristics between adult patients with ADHD and HCs and to classify them via machine learning measures.

## Materials and methods

### Participants

A total of 79 subjects aged 18–45 years participated in this study. Subjects included patients with ADHD (*n* = 34, male: 28, female: 6, mean age: 24.76 ± 7.02 years; range: 19–45 years) and healthy controls (*n* = 45, male: 36, female: 9, mean age: 25.51 ± 5.48 years; range: 18–37 years). Participants with ADHD were enrolled from the Department of Psychiatry at Soonchunhyang University Cheonan Hospital, Korea. All psychiatric evaluations were conducted by a board-certified psychiatrist specializing in adult ADHD using the full criteria for ADHD in accordance with the DSM-V. Patients who had mental retardation or alcohol abuse, undergone electroconvulsive therapy, or suffered head injury were not included. All patients with ADHD were drug-naïve. Forty-five non-smoking HCs were enrolled by the local community via newspapers and posters. We excluded participants with any axis I or II comorbid psychiatric diagnosis or any history of neurological diseases from the initial screening interviews. Participants had normal hearing ability confirmed by the 512 Hz tuning fork test [27], and all were identified as right-handed. The Institutional Review Board and Ethics Committee of Soonchunhyang University Cheonan Hospital approved the study and all experimental protocols (IRB number: 2019-05-004). The study was conducted according to the approved guidelines. Informed consent was acquired from all study participants.

### Psychological measures

All participants were assessed for ADHD symptoms using the Korean version of the Adult ADHD self-report scales (ASRS) [28]. The ASRS is a widely used self-reporting scale with 18 items scored on a 5-point Likert scale to screen for ADHD in the general population [29]. It evaluates ADHD symptoms based on the DSM-IV criteria for ADHD over the past six months. Inattention (ASRS inattention score, ASRS-I) and hyperactivity scores (ASRS hyperactivity score, ASRS-H) were calculated separately. The Korean version of the ASRS shows good sensitivity and specificity [28].

### Data acquisition and analysis

EEG data were recorded by a NeuroScan SynAmps2 amplifier (Compumedics USA, Charlotte, NC, USA) with 62 Ag-AgCl channels mounted on a Quik Cap, using an extended 10–20 placement scheme. The ground channel was placed on the forehead, and the physically linked reference channel was attached to both mastoids. Vertical electrooculogram (EOG) channels were located above and below the left eye. Horizontal EOG channels were placed at the outer canthus of each eye. The impedance was maintained below 5 kΩ. The EEG data were obtained with a band-pass filter with cutoff frequencies ranging from 0.1 to 100 Hz at a 1000 Hz sampling rate.

The acquired EEG data were preprocessed by CURRY 8 (Compumedics USA, Charlotte, NC, USA) and MATLAB R2018b (MathWorks, Natick, MA, USA). Gross artifacts were rejected from visual inspection of an experienced person without any prior knowledge concerning the origin of the data. Artifacts related to eye movements or eye blinks were corrected using a covariance- and regression-based mathematical procedure implemented in the preprocessing software [30]. The data were filtered using a 1–30 Hz band-pass filter. Then, the data were epoched from 100 ms pre-stimulus to 600 ms post-stimulus. For baseline correction, the epochs were deducted from the mean value of the pre-stimulus interval. If there were any remaining epochs containing significant physiological artifacts (amplitude exceeding ±75 μV) in any of the 62 channel sites, they were rejected from further analyses. For the ERP analysis, only artifact-free epochs were averaged along trials and participants.

EEG recordings and stimulus presentation onset were synchronized by E-prime (Psychology Software Tools, Pittsburgh, PA, USA). The auditory stimuli composed of sounds at 1000 Hz and 85 dB SPL. The participants were instructed to focus their attention on a picture book called “Where’s Wally?” without paying attention to the auditory stimuli. To obtain the MMN wave, the ERP wave derived from standard stimuli was subtracted by the ERP wave from deviant stimuli for each participant.

Standard stimuli lasting 50 ms were presented, randomly interspersed with deviant stimuli lasting 100 ms (90% and 10% probabilities, respectively). There were a total of 750 auditory stimuli with a 500 ms inter-stimulus interval. These stimuli were presented by MDR-D777 headphones (Sony, Tokyo, Japan). The experiment took about 10 min to complete.

MMN amplitude was measured as the peak voltage between 130 and 280 ms at nine channels (F3, Fz, F4, FC3, FCz, FC4, C3, Cz, and C4) according to previous studies revealing that the frontocentral electrodes show larger MMN amplitudes [31–33]. The time range for MMN peak amplitudes was on the basis of the grand-averaged waveforms at FCz channel. The number of epochs for standard and deviant stimuli in the analysis did not significantly differ between patients with ADHD and HCs (standard stimuli: 598.59 ± 66.12 *vs*. 609.18 ± 48.56, *p* = 0.414; deviant stimuli: 66.91 ± 7.46 *vs*. 67.71 ± 5.14, *p* = 0.575).

### Source imaging

In order to estimate the cortical distribution of the standardized source current density for MMN activity, standardized low-resolution brain electromagnetic tomography (sLORETA) was employed. sLORETA has been widely utilized as a representative source-imaging method to solve the EEG inverse problem [34]. It assumes that the source activity of a voxel is similar to that of the neighboring voxels when computing a particular solution, and applies a proper standardization for the current density. The lead field matrix was calculated using a realistic head model which was segmented according to the Montreal Neurological Institute (MNI) 152 standard template, wherein the three-dimensional solution space was confined to only the cortical gray matter and hippocampus [35]. The three-dimensional solution space consisted of 6239 voxels with a 5-mm resolution. Anatomical labels including the Brodmann areas were provided with a proper transformation from the MNI to Talairach space [36].

The MMN source image was analyzed between 130 and 280 ms after stimulus onset. The comparison of sLORETA images between adult patients with ADHD and HCs for MMN was conducted by a statistical non-parametric mapping method (SnPM) carried out in the sLORETA software. The estimated voxel activation was averaged across the calculated time frame and tested with a voxel-by-voxel independent *t*-test for the 6239 voxels, followed by a randomization test (*n* = 5000) for correcting multiple comparisons.

### Statistical analysis

Differences of age, education years, and psychological characteristics (ASRS) between adult patients with ADHD and HCs were compared using independent *t*-tests. A chi-squared test was used to analyze between-group difference in sex ratio. The significance level was *p* < 0.05 (two-tailed). A multivariate analysis of variance (MANOVA) was performed to evaluate differences in MMN amplitudes and latencies at frontocentral electrodes and MMN source activities between the two groups, with education years as a covariate. An adjusted *p*-value of 0.05/52 = 0.000962 (18 features from sensor level and 34 features from source level) by Bonferroni corrections were used to control for multiple comparisons. Effect sizes were represented as partial eta squared (*η*^2^). A partial Pearson’s correlation analysis was conducted between MMN features from sensor and source levels and ADHD symptom scores, with years of education controlled for. After the correlation analysis, an adjusted *p*-value of 0.05/(3*27) = 0.000617 (27 significant MMN features with three ASRS scores) by Bonferroni corrections were applied. Statistical analyses were carried out using SPSS 21 (SPSS, Inc., Chicago, IL, USA).

### Feature selection and classification

We discriminated between adult ADHD patients and HCs using the MMN sensor and source activities to check their potential usability as biomarkers. To find optimal features for discriminating the two groups, both MMN features from sensor and source levels were used as follows (Table 1): sensor-level (18 features), each of the nine frontocentral MMN amplitudes and latencies (F3, Fz, F4, FC3, FCz, FC4, C3, Cz, and C4); source-level (34 features), MMN source activities in 34 brain regions (frontal areas: superior frontal gyrus, middle frontal gyrus, medial frontal gyrus, inferior frontal gyrus, orbital gyrus, subcallosal gyrus, rectal gyrus; temporal areas: superior temporal gyrus, middle temporal gyrus, inferior temporal gyrus, transverse temporal gyrus; limbic areas; anterior cingulate cortex, insula, extra nuclear, parahippocampla gyrus, uncus, cingulate gyrus). We selected these brain regions on the basis of the former neuroimaging and ERP source localization studies for MMN generator [37–44], and the results of MMN source activities showing significant differences between the two groups in our data.

**Table 1:** All MMN features including sensor and source levels for classification between adult ADHD patients and healthy controls.

Classification features	
Sensor level (18 features)	Source level (34 features)
MMN peak amplitude	MMN source activity
FC3 peak amplitude	Frontal lobe
FCz peak amplitude	Bilateral superior frontal gyrus
FC4 peak amplitude	Bilateral middle frontal gyrus
F3 peak amplitude	Bilateral medial frontal gyrus
Fz peak amplitude	Bilateral inferior frontal gyrus
F4 peak amplitude	Bilateral orbital gyrus
C3 peak amplitude	Bilateral subcallosal gyrus
Cz peak amplitude	Bilateral rectal gyrus
C4 peak amplitude	Temporal lobe
MMN peak latency	Bilateral superior temporal gyrus
FC3 peak amplitude	Bilateral middle temporal gyrus
FCz peak latency	Bilateral inferior temporal gyrus
FC4 peak latency	Bilateral transverse temporal gyrus
F3 peak latency	Limbic lobe
Fz peak latency	Bilateral anterior cingulate cortex
F4 peak latency	Bilateral insula
C3 peak latency	Bilateral extra nuclear
Cz peak latency	Bilateral parahippocampal gyrus
C4 peak latency	Bilateral uncus
	Bilateral cingulate gyrus

*ADHD* attention-deficit/hyperactivity disorder, *MMN* mismatch negativity.

Classification was performed using a linear support vector machine classifier with the cost set as 1 [45–47] and the classification accuracy was evaluated using a leave-one-out cross-validation method for each feature set. Linear support vector machine has been widely applied in multivariate pattern analysis with its high accuracy, generalization, and interpretability [48–50]. Many studies have used the leave-one-out cross-validation method claiming that it is more appropriate for small data since more data can be trained for a classification model and that it imitates clinical setting where clinicians can learn from large data and apply the findings to new each case [51–53]. In order to decrease the computational cost and prevent potential overfitting by the large number of features, the Fisher score was used for feature selection [54, 55]. For each cross-validation, the Fisher score was used to select the best feature subset for the current training dataset. A higher Fisher score for each feature represents better separability between the two groups. Different numbers of features with higher Fisher scores ranging from 1 to 20 were respectively examined for classification between the two groups [56]. Finally, the classification performances including accuracy, sensitivity, and specificity were averaged in the leave-one-out cross-validation.

In addition, 1000 times permutation test (group label permutation) were performed to assess the statistical significance of our classification accuracy [47, 57, 58]. We labeled each participant to two groups randomly, trained and tested the support vector machine classifier with this random labeling, and calculated the accuracy from the classification model. A MATLAB toolbox, Pattern Recognition Tools 5 (http://37steps.com) for the machine learning analyses.

## Results

### Demographic and psychological characteristics

Table 2 presents the demographic and psychological characteristic comparison between the patients with ADHD and HCs. There was a significant difference in education years. HCs showed significantly higher education years than patients with ADHD (*p* < 0.001). In addition, there were significant differences in ASRS scores between the two groups, such that the patients with ADHD showed significantly higher ASRS (*p* < 0.001) and its subscales, including inattention (*p* < 0.001) and hyperactivity (*p* < 0.001).

**Table 2:** Demographic characteristics of study participants.

	ADHD (*N* = 34)	HC (*N* = 45)	*P*
Age (years)	24.76 ± 7.02	25.51 ± 5.48	0.597
Sex			0.792
Male	28 (82.4)	36 (80.0)	
Female	6 (17.6)	9 (20.0)	
Education (years)	12.82 ± 1.40	14.36 ± 2.31	<0.001
Adult ADHD Self-Report Scale (ASRS)	42.12 ± 11.13	10.60 ± 8.98	<0.001
Inattention	24.21 ± 5.84	7.27 ± 5.61	<0.001
Hyperactivity	17.91 ± 6.15	3.33 ± 4.09	<0.001

*ADHD* attention-deficit/hyperactivity disorder, *HC* healthy control.

### Mismatch negativity

**Fig. 1:**
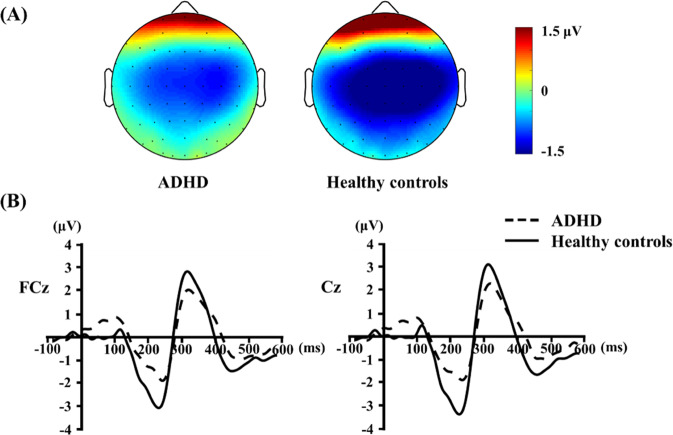
Topographical distributions and grand-average waveforms for mismatch negativity (MMN) in adult patients with ADHD and healthy controls. (**A**) Topographic maps of MMN, and (**B**) Grand average of MMN waveforms at FCz and Cz electrodes.

The patients with ADHD showed significantly reduced MMN peak amplitudes compared to HCs at the FCz (*p* < 0.001), FC4 (*p* < 0.001), C3 (*p* < 0.001), Cz (*p* < 0.001), and C4 (*p* < 0.001) electrodes. However, there was no significant difference in MMN latency between the two groups. Figure 1 shows the grand-average waveforms and topographical distributions for MMN in each group.

### Source analysis

**Fig. 2:**
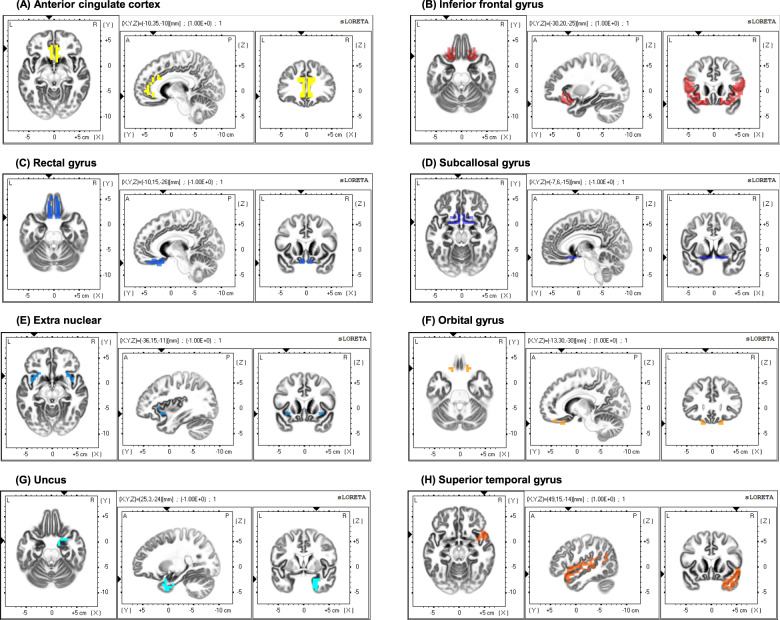
Brain regions showing the best classification performances from the source-level feature set between adult ADHD patients and healthy controls. (**A**) Anterial cingulate cortex, (**B**) Inferior frontal gyrus, (**C**) Rectal gyrus, (**D**) Subcallosal gyrus, (**E**) Extra nuclear, (**F**) Orbital gyrus, (**G**) Uncus, and (**H**) Superior temporal gyrus.

Adult patients with ADHD revealed significantly decreased MMN source activities compared to HCs in the right middle frontal gyrus, right medial frontal gyrus, bilateral anterior cingulate cortex, bilateral inferior frontal gyrus, right superior temporal gyrus, right inferior temporal gyrus, bilateral insula, bilateral extra nuclear, bilateral orbital gyrus, bilateral parahippocampal gyrus, bilateral rectal gyrus, bilateral subcallosal gyrus, and bilateral uncus (*p* < 0.001; Table 3, Fig. 2).

**Table 3:** Mean values and standard deviations of MMN source activities in brain regions showing the best classification performances from the source-level feature set between adult ADHD patients and healthy controls.

Region of Interest	ADHD (*N* = 34)	HC (*N* = 45)	Effect size (*η*^2^)	*P*
*Left*				
Anterior cingulate cortex	2.74 ± 1.96	6.36 ± 3.54	0.227	<0.001
Inferior frontal gyrus	2.79 ± 1.62	5.46 ± 2.87	0.191	<0.001
Rectal gyrus	4.46 ± 3.59	10.53 ± 6.17	0.216	<0.001
Subcallosal gyrus	2.22 ± 1.66	5.45 ± 2.97	0.263	<0.001
Extra nuclear	2.16 ± 1.41	4.20 ± 2.23	0.188	<0.001
Orbital gyrus	5.84 ± 4.65	12.98 ± 7.99	0.181	<0.001
*Right*				
Anterior cingulate cortex	2.72 ± 1.92	6.16 ± 3.46	0.208	<0.001
Inferior frontal gyrus	2.64 ± 1.78	5.15 ± 2.23	0.256	<0.001
Rectal gyrus	4.68 ± 3.80	11.19 ± 6.44	0.222	<0.001
Subcallosal gyrus	1.99 ± 1.52	5.13 ± 2.65	0.301	<0.001
Extra nuclear	2.08 ± 1.50	4.44 ± 2.09	0.298	<0.001
Orbital gyrus	5.71 ± 4.73	12.90 ± 7.64	0.192	<0.001
Uncus	2.13 ± 1.69	5.19 ± 2.99	0.248	<0.001
Superior temporal gyrus	1.74 ± 1.10	3.19 ± 1.43	0.237	<0.001

*ADHD* attention-deficit/hyperactivity disorder, *HC* healthy control, *MMN* mismatch negativity.

### Correlation between MMN characteristics and ADHD symptoms

**Fig. 3:**
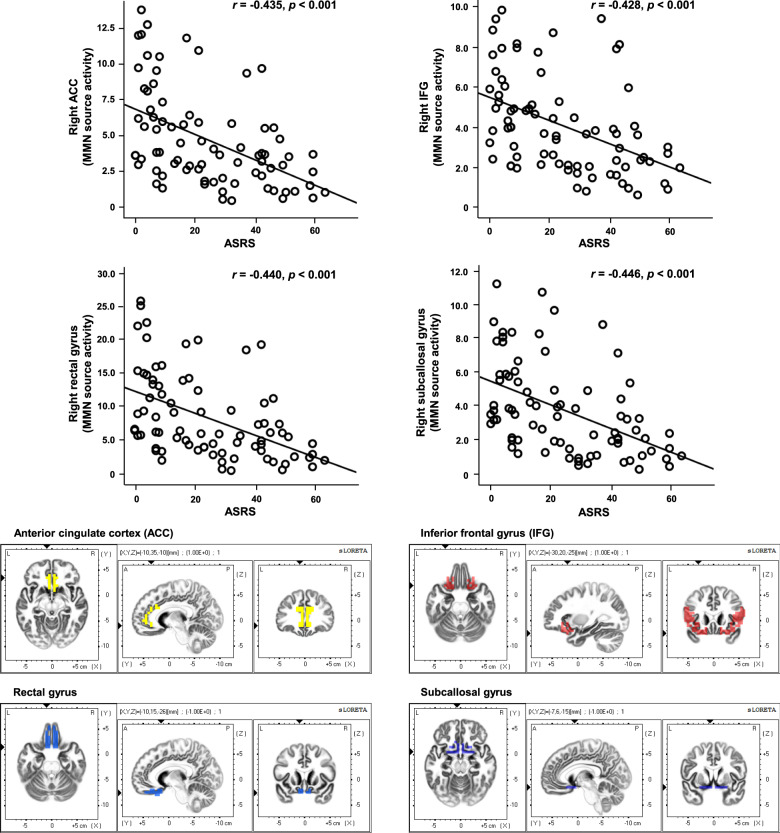
Correlation between mismatch negativity (MMN) characteristics and ADHD symptoms. Correlations between MMN source activities in anterior cingulate cortex, inferior frontal gyrus, rectal gyrus, and subcallosal gyrus and ADHD symptom scores in all participants.

Correlation analysis detected significant correlations between the MMN source activities and ADHD symptoms in the study population: (1) ASRS: left anterior cingulate cortex (*r* = −0.427, *p* < 0.001), right anterior cingulate cortex (*r* *=* −0.435, *p* < 0.001), right inferior frontal gyrus (*r* *=* −0.428, *p* < 0.001), right superior temporal gyrus (*r* *=* −0.385, *p* < 0.001), right extra nuclear (*r* *=* −0.413, *p* < 0.001), left orbital gyrus (*r* *=* −0.401, *p* < 0.001), right orbital gyrus (*r* *=* −0.428, *p* < 0.001), left rectal gyrus (*r* *=* −0.418, *p* < 0.001), right rectal gyrus (*r* *=* −0.440, *p* < 0.001), left subcallosal gyrus (*r* *=* −0.408, *p* < 0.001), right subcallosal gyrus (*r* *=* −0.446, *p* < 0.001), right uncus (*r* *=* −0.401, *p* < 0.001); (2) inattention: left anterior cingulate cortex (*r* *=* −0.417, *p* < 0.001), right anterior cingulate cortex (*r* *=* −0.435, *p* < 0.001), right inferior frontal gyrus (*r* *=* −0.424, *p* < 0.001), right extra nuclear (*r* *=* −0.394, *p* < 0.001), left orbital gyrus (*r* *=* −0.396, *p* < 0.001), right orbital gyrus (*r* *=* −0.434, *p* < 0.001), left rectal gyrus (*r* *=* −0.412, *p* < 0.001), right rectal gyrus (*r* *=* −0.440, *p* < 0.001), left subcallosal gyrus (*r* *=* −0.393, *p* < 0.001), right subcallosal gyrus (*r* *=* −0.430, *p* < 0.001), right uncus (*r* *=* −0.388, *p* < 0.001); (3) hyperactivity: left anterior cingulate cortex (*r* *=* −0.413, *p* < 0.001), right anterior cingulate cortex (*r* *=* −0.409, *p* < 0.001), right inferior frontal gyrus (*r* *=* −0.407, *p* < 0.001), right extra nuclear (*r* *=* −0.410, *p* < 0.001), left orbital gyrus (*r* *=* −0.384, *p* < 0.001), right orbital gyrus (*r* *=* −0.396, *p* < 0.001), left rectal gyrus (*r* *=* −0.401, *p* < 0.001), right rectal gyrus (*r* *=* −0.413, *p* < 0.001), left subcallosal gyrus (*r* *=* −0.401, *p* < 0.001), right subcallosal gyrus (*r* *=* −0.438, *p* < 0.001), right uncus (*r* *=* −0.393, *p* < 0.001) (Fig. 3). There was no significant correlation between MMN sensor-level features and ADHD symptom scores.

### Classification results

Table 4 presents the best classification performances between adult ADHD patients and HCs for three different feature sets (sensor-level, source-level, and both sensor and source levels). For the sensor-level feature set, the best classification accuracy was 78.48% with three features: MMN amplitudes at FCz, FC4, and Cz electrodes. In terms of source-level feature set, the best classification accuracy was 81.01% with 14 features, MMN source activities in the bilateral anterior cingulate cortex, bilateral inferior frontal gyrus, bilateral rectal gyrus, bilateral subcallosal gyrus, bilateral extra nuclear, bilateral orbital gyrus, right uncus, and right superior temporal gyrus. For both sensor and source levels, the best classification accuracy was 81.01% with 16 features, MMN amplitude at FC4 and MMN source activities in the bilateral anterior cingulate cortex, bilateral inferior frontal gyrus, bilateral rectal gyrus, bilateral subcallosal gyrus, bilateral extra nuclear, bilateral orbital gyrus, bilateral uncus, and right superior temporal gyrus.

**Table 4:** The best classification performances (%) of adult ADHD patients and healthy controls for three different feature sets (sensor-level, source-level, and both sensor- and source-levels).

	ADHD vs. HC		
	Accuracy	Sensitivity	Specificity
Sensor level	78.48	70.59	84.44
Source level	81.01	82.35	80.00
Sensor level + source level	81.01	79.41	82.22

*ADHD* attention-deficit/hyperactivity disorder, *HC* healthy control.

The permutation test results demonstrated that our classification accuracies were significant. The classification accuracies from the original data were higher than all the 1000 classification accuracies from the permutation test for the three different feature sets (mean ± 2 standard deviation; sensor-level: 54.92 ± 9.60%, source-level: 52.47 ± 13.96%, both sensor and source levels: 52.03 ± 15.21%).

## Discussion

In this study, we investigated MMN abnormalities, indicative of pre-attentive central processing of auditory change detection, in drug-naive adult patients with ADHD and differentiated ADHD patients and HCs based on machine learning methods. Our major findings are as follows. First, the patients with ADHD showed significantly reduced MMN amplitudes at the frontocentral electrodes compared to HCs. Second, the patients exhibited significantly decreased MMN source activities in the frontal, temporal, and limbic regions. Third, MMN source activities were negatively correlated with ADHD symptom scores in the study population. Fourth, the best classification performance for adult ADHD patients and HCs was an 81.01% accuracy, an 82.35% sensitivity, and an 80.00% specificity from the MMN source activity features.

The MMN amplitudes were attenuated in adult patients with ADHD compared to HCs at the frontocentral electrodes. Although there has been no previous study exploring MMN in adult patients with ADHD, our results are comparable to those of previous studies showing decreased MMN amplitudes in children and adolescent patients with ADHD [7, 17, 59]. Given the notion that MMN reflects pre-attentive detection related to cognitive status [9, 10], the attenuated MMN amplitudes suggest that adult patients with ADHD also suffer from dysfunctional pre-attentive processes. Moreover, MMN has been thought to reflect information processing more accurately than the P300 ERP component, and has been closely related to attention deficit [59]. Although the MMN is not subject to conscious control, the changes in MMN in adult patients with ADHD might reflect the dysfunctional attentional process of ADHD [60, 61]. Impairments in deviance detection might cause significant dysfunctions in higher-order cognitive functioning, affecting the inattentive and hyperactive symptoms of patients with ADHD.

In this study, adult patients with ADHD showed significantly reduced MMN source activities compared to HCs in the frontal lobe (right middle frontal gyrus, right medial frontal gyrus, bilateral inferior frontal gyrus, bilateral orbital gyrus, bilateral subcallosal gyrus, bilateral rectal gyrus), temporal lobe (right superior temporal gyrus, right inferior temporal gyrus), and limbic lobe (bilateral anterior cingulate cortex, bilateral insula, bilateral extra nuclear, bilateral parahippocampal gyrus, bilateral uncus). In addition, reduced source activities in the bilateral anterior cingulate cortex, right inferior frontal gyrus, right superior temporal gyrus, right extra nuclear, bilateral orbital gyrus, bilateral rectal gyrus, bilateral subcallosal gyrus, and right uncus were negatively correlated with the ADHD symptom scales in the study population.

In general, auditory MMN is produced in the primary auditory cortex and adjacent areas of the superior temporal lobe [42, 62]. The frontal areas, including the anterior cingulate cortex and middle and inferior frontal gyrus, are also considered MMN generators [39, 42, 63–65]. Temporal generators are related to auditory feature analysis and deviance detection, and frontal generators with involuntary switching of attention toward changes in the auditory environment [38, 66]. In particular, frontal generators have been associated with a cognitive role or comparator-based mechanism of MMN [67–69].

Regions in which we found dysfunctional MMN source activities and significant symptomatic correlations were areas of focus in several previous ADHD studies. According to previous neuroimaging studies, the development of ADHD was closely associated with impaired frontal lobe function [70, 71]. Altered neurochemistry in the anterior cingulate cortex is thought to be an important cause of behavioral symptoms of ADHD [72]. Structural abnormalities such as smaller volume and reduced cortical thickness in the anterior cingulate cortex have been observed in both children and adult patients with ADHD than in HCs [73–77]. In a variety of cognitive tasks, patients with ADHD have shown abnormal performance that might be specifically linked to structural and/or functional anomalies in the anterior cingulate cortex [78, 79].

In addition, several studies have highlighted that the inferior frontal gyrus is crucial for inhibiting behavioral responses related to impulsivity and hyperactivity [80, 81]. Opitz et al. [82] suggested that the inferior frontal gyrus might be associated with an involuntary amplification or contrast enhancement mechanism, regulating the auditory change detection system. Bayard et al. reported that ADHD symptoms were negatively correlated with gray matter volume in the bilateral inferior frontal cortex [83]. In terms of the orbitofrontal region, including the orbital gyrus, cortical thinning and volumetric reduction of this region have been reported in adult patients with ADHD [74, 84]. In addition, the orbitofrontal region was revealed to be primarily related to emotional instability and impulsivity in ADHD [84, 85].

Furthermore, the insula is known to play a generic role in updating information and cognitive control, including attention allocation to behaviorally relevant salient stimuli [86–88]. The functions mediated by the insula are consistently impaired in ADHD, such as cognitive control, sustained attention, and saliency detection [89, 90]. One previous volumetric study revealed that youths with ADHD showed a bilateral reduction in the insula region associated with attention problems and inhibition [91]. The superior temporal region has been reported to be abnormal in ADHD. Imaging studies using oddball involuntary attention tasks showed reduced activation in the bilateral superior temporal gyrus in adolescents with ADHD [92, 93]. Previous studies have demonstrated that there are delays in cortical maturation in the bilaterally superior temporal gyrus of ADHD patients that could affect the control of attention [94, 95].

These abnormal regions belonging to frontal, temporal, and limbic lobes are highly related to the pathophysiology of ADHD. The previous findings support our results regarding altered source activation for MMN in adult ADHD patients and correlations between source activation and ADHD symptom scores. Decreased MMN source activation in adult patients with ADHD could be interpreted as decreased efficiency of automatically regulated attentional processes and increased demand for more effort and resources.

We applied machine learning methods to classify adult ADHD patients and HCs using sensor- and source-level features extracted from MMN ERP data. The best classification performance was achieved when source-level features were used (accuracy: 81.01%, sensitivity: 82.35%, sensitivity: 80.00%). According to our results, the source-level features from MMN might play a more important role in the classification of ADHD patients and HCs than the sensor-level features. This might be due to the notion that the shortcoming of sensor-level features could be supplemented by source-level features such as low spatial resolution originating from volume conduction and poor signal-to-noise ratio [96–98]. Thus, the improved spatial information from the source-imaging method might contribute to the enhanced classification performance. When the best classification performance was achieved in the classification of the patients and HCs using source-level features, 14 regions were selected (bilateral anterior cingulate cortex, bilateral inferior frontal gyrus, bilateral rectal gyrus, bilateral subcallosal gyrus, bilateral extra nuclear, bilateral orbital gyrus, right uncus, and right superior temporal gyrus). MMN source activation in the frontal, temporal, and limbic lobes was dysfunctional in adult ADHD patients. Our results suggest that the reduced MMN activations in these regions could be utilized as important neuromarkers for classifying adult ADHD patients from HCs and could help to elucidate the neural mechanism of adult ADHD during pre-attentive processing.

Moreover, when we used both sensor- and source-level features for the classification of adult ADHD patients and HCs, we obtained a classification accuracy of 81.01%, which was the same value as that from source-level features. However, the sensitivity was higher for source-level features (82.35%) than for both sensor- and source-level features (79.41%). In the clinical setting, the value of sensitivity, referring to the ability to correctly identify patients with the disease, could be considered more important [99]. Therefore, although the accuracies from source-level features and both sensor- and source-level features were the same, the source-level features might play a more crucial role in the clinical setting as biomarkers for adult ADHD patients.

In this study, a number of MMN features including sensor and source levels showed significant between-group differences based on the univariate analysis method (*p* < 0.001). The statistics for the univariate analyses were calculated based on the differences of mean and standard deviation between two groups. Thus, even though the data distribution of the two groups from the features was overlapped, the features could show significant between-group differences with their mean and standard deviation differences. However, to achieve higher classification performances using machine learning, the distribution of data between two groups should be less overlapped. We achieved the classification accuracy of 81.01% between adult ADHD patients and HCs using the source-level feature set. This classification accuracy was acceptable and we found that the accuracy was significantly based on the permutation test. Since our study was the first to investigate MMN changes in adult patients with ADHD, further studies with a larger number of adult patients with ADHD are warranted to verify our findings.

This study has several limitations. First, when interpreting the results, the relatively small sample size should be considered. Further studies are warranted to validate the findings with larger samples. Second, the patient group and HCs were not matched for education years. However, the difference in education years between the groups has been accepted in recent studies [100], and we controlled the variable as a covariate in our statistical analyses. Third, we did not implement individual head models for EEG source imaging. Despite the above limitations, this study is noteworthy as the first attempt to investigate the differences in MMN activities in adult patients with ADHD and HCs. Altered MMN features at both the sensor and source levels were found in ADHD patients compared to HCs, and significant correlations between MMN source activities and ADHD symptom scores were observed. Furthermore, we achieved acceptable classification performances using MMN features for discriminating between the groups. Our results showed the possibility of MMN features as biomarkers for adult ADHD patients, suggesting that the MMN might offer additional useful information for detecting patients in the clinical setting. As a future study, we will attempt to improve classification performances based on deep-learning techniques with larger sample sizes.
